# Hepatitis C virus spread from HIV-positive to HIV-negative men who have sex with men

**DOI:** 10.1371/journal.pone.0190340

**Published:** 2018-01-02

**Authors:** Caroline Charre, Laurent Cotte, Rolf Kramer, Patrick Miailhes, Matthieu Godinot, Joseph Koffi, Caroline Scholtès, Christophe Ramière

**Affiliations:** 1 Laboratoire de Virologie, Hôpital de la Croix-Rousse, Hospices Civils de Lyon, Lyon, France; 2 Centre International de Recherche en Infectiologie (CIRI) (Inserm U1111, CNRS UMR 5308), Lyon, France; 3 University of Lyon, Université Claude Bernard Lyon 1, Villeurbanne, France; 4 Service des Maladies Infectieuses, Hôpital de la Croix-Rousse, Hospices Civils de Lyon, Lyon, France; 5 INSERM U1052, Cancer Research Center of Lyon (CRCL), UMR_S1052, Lyon, France; 6 European Public Health Microbiology Training Programme (EUPHEM), European Centre for Disease Prevention and Control, Stockholm, Sweden; 7 Centre for Clinical Research, Department of Hepatology, Groupement Hospitalier Nord, Hospices Civils de Lyon, Lyon, France; Centre de recherche du CHUM, CANADA

## Abstract

The aim of this study was to evaluate the potential transmission of HCV strains between HIV-positive men who have sex with men (MSM) and HIV-negative MSM. Since 2000, an ongoing epidemic of HCV infections is observed among HIV-positive MSM in high-income countries. However, HCV infections in HIV-negative MSM are investigated to a lesser extent due to the lack of follow-up in this population and only limited information is available on the risk of HCV transmission between HIV-positive MSM and HIV-negative MSM. We enrolled 49 MSM of which 43 were HIV-positive and 6 HIV-negative, including 4 being enrolled or waiting for enrolment in a preexposure prophylaxis (PrEP) program. All patients were diagnosed with acute HCV infection at the Infectious Disease Unit at the Hospices Civils de Lyon from 2014 to 2016. Risk factors for HCV infection were similar in both groups and included IV or nasal drug use, and rough sex practices. Typing and phylogenetic cluster analysis of HCV variants were performed by NS5B sequencing. Several clusters of infections were identified (genotype 1a: 3 clusters and 1 pair; genotype 4d: 1 cluster and 2 pairs), suggesting that several transmission events occurred within the study population. Every HCV strain identified in HIV-negative MSM was included in a cluster with HIV-positive MSM. Chronological analysis of contagiousness suggested the transmission of HCV from HIV-positive to HIV-negative patients. We conclude that recommendations for HCV surveillance should not be confined to HIV-positive MSM but should be extended to HIV-negative MSM with similar risk factors.

## Introduction

Since 2000, acute hepatitis C infections in human immunodeficiency virus (HIV)-positive men who have sex with men (MSM) are increasing in many regions worldwide [1], such as Europe [2–4], the United States [5,6], Australia [7] and China [8]. A large proportion of infections were attributable to high-risk practices, including unprotected mucosally-traumatic sex (rough sex) and sharing of recreational drugs via nasal, anal or intravenous routes [3,9,10].

The hepatitis C virus (HCV) epidemic has been well described among HIV-positive MSM with an estimated incidence rate increasing from 0.42/100 person-years in 1991 to 1.34/100 in 2010 [1]. Reinfections rate was consistently higher in HIV-positive MSM than the incidence rate for a first infection, which has been assumed to reflect a heterogeneous risk among this population, some HIV-positive MSM having a high-risk of HCV due to their high-risk practices, whilst the risk of HCV in the absence of high-risk practices was lower (Virlogeux et al., oral communication at CROI 2017). At that time, the spread of hepatitis C among HIV-negative MSM appeared limited [11,12], with a meta-analysis showing a pooled incidence rate of 0.15 per 100 person-years in this population [13]. The lower incidence rate of HCV infection in HIV-negative MSM may partly be explained by the fact that HIV infection increases HCV susceptibility and transmission [14]. Indeed, HIV-associated disruption of mucosal epithelial junctions might facilitate the penetration and dissemination of HCV [15,16]. Likewise, MSM with HIV infection have higher seminal HCV loads than HIV-negative MSM which is known to promote HCV transmission [17]. Moreover, unprotected anal sex with partners of similar HIV status (serosorting) may also contribute to the imbalanced incidence rate of HCV infection between HIV-positive and HIV-negative MSM [18]. However, the lack of regular HCV screening and transaminase assessment in HIV-negative MSM may contribute to underestimate acute HCV infections in this population. The relation between an increasing incidence of acute HCV in HIV-negative MSM and the development of pre-exposure prophylaxis (PrEP) programs for the prevention of HIV infection in high-risk MSM remains controversial [19]. On the one hand, one can consider that PrEP may lead to an increase in unprotected anal intercourse regardless of HIV status and thus lead to higher HCV transmission between HIV-positive and HIV-negative MSM. On the other hand, high-risk practices in PrEP users appeared similar to those observed in HIV-positive MSM, thus no difference in terms of HCV incidence might be expected. Indeed, HCV incidence rates among PrEP users appear in the same range than those observed in HIV-positive MSM at the same time (0.7/100 person-years in years 2011–2014, to 1.2/100 person-years in 2014) [20,21]. One might therefore suspect that HCV has spread, over the last years, from HIV-positive to HIV-negative MSM, sharing the same high-risk practices, but only limited information is currently available regarding this risk of transmission.

The aim of this study was to evaluate the potential transmission of HCV strains between HIV-positive and HIV-negative MSM. To assess this hypothesis, we performed a phylogenetic analysis of acute HCV infections in MSM regardless of HIV status and attempted to determine the chronology of infections within clusters.

## Material and methods

### Study population

The study cohort included all MSM patients who were diagnosed with acute HCV infection at the Infectious Disease Department of the Hospices Civils de Lyon, France, and for whom NS5B sequencing was performed between January 2014 and December 2016. Data on drug use patterns and risk behaviours were collected. HCV isolated from 42 non-MSM, HIV-negative, male patients of similar age (genotype 1a: 30; genotype 4d: 12) were analysed by NS5B sequencing at the same time for phylogenetic analysis. This study was conducted in accordance with French ethics regulations. All patients gave their written inform consent to allow the use of their personal clinical data. The study was approved by the Ethics Committee of Hospices Civils de Lyon.

### Case finding

HCV infections were diagnosed during: i) regular follow-up visits for HIV-positive patients and ii) during visits for PrEP or for sexually transmitted infections (STI) for HIV-negative patients. The time of acute HCV infections was based on the first occurrence of: i) seroconversion for anti-HCV antibodies or ii) detection of HCV RNA in plasma before seroconversion or iii) concomitant detection of anti-HCV antibodies and HCV RNA in a context of elevated transaminases with a history of recent HCV exposure and negative previous serology and normal transaminases.

The period of contagiousness for HCV was defined by the presence of detectable HCV RNA in plasma during follow-up.

### Risk practices

Bacterial sexually transmitted infections (syphilis, gonorrhoea, chlamydia infection) at the time of HCV diagnosis, the use of IV or nasal recreational drugs and rough sex practices such as sex in group, fisting or sharing sex toys were recorded from patients file.

### HCV testing and sequencing

HCV RNA was detected and quantified using the Abbott RealTime HCV assay (Abbott Molecular, Rungis, France).

The NS5B fragment of HCV was amplified between nucleotides 8256 and 8644 by RT-PCR as previously described [22] and sequenced using the Sanger method. Electrophoresis and data collection were performed on a GenomeLab^™^ GeXP Genetic Analyzer (Beckman Coulter). Consensus sequences were assembled and analysed using the GenomeLab^™^ sequence analysis software. The genotype of each sample was determined by comparing its sequence with HCV reference sequences [23] obtained from GenBank.

### Phylogenetics

Phylogenetic trees for genotype 1a and genotype 4d sequences were constructed using MetaPIGA v.3.100 software. The Maximum Likelihood criterion was applied using the Hasegawa-Kishino-Yano (HKY) substitution model [24]. MetaPIGA calculations were stopped when the mean relative error of 10 consecutive consensus trees stayed below 5% using trees sampled every five generations. Consensus trees were visualized in Figtree v.1.4.3 (http://tree.bio.ed.ac.uk/software/figtree/). Reference strains for genotype 1a (Genbank accession numbers AF009606, EF407457, HQ850279, M62321 and M67463) and for genotype 4d (Genbank accession numbers DQ418786, FJ462437 and EU392172) were chosen for comparison.

Identification of transmission clusters was performed using Cluster Picker software with a support threshold of 0.9 and a genetic distance threshold of 0.045 [25]. Transmission “clusters” and “pairs” were defined as phylogenetic clades with n≥3 and n = 2 sequences respectively.

### Statistics

Considering the limited number of HIV-negative patients infected with HCV in the study, no statistical methods were applied to compare characteristics between HIV-positive and HIV-negative patients.

### Nucleotide accession numbers

All HCV NS5B sequences isolated in MSM and non-MSM patients reported in this study were submitted to the GenBank database. The list of Genbank accession numbers for all sequences is provided in S1 Table.

## Results

### Study population characteristics

The study population included 43 HIV-positive MSM and 6 HIV-negative MSM infected with HCV. Patient characteristics are described in Table 1. Whilst no comparison could be done due to the limited number of patients, HIV-negative patients appeared younger than HIV-positive patients (39.5 years vs 48 years). Most of the HIV-positive patients were on antiretroviral treatment, with an undetectable HIV-RNA and CD4 cells count over 500/mm^3^. Four out of 6 HIV-negative MSM were PrEP users but only 1 patient was receiving PrEP at the time of HCV diagnosis. For each group, reported rates of risk behaviour were high. Indeed, at least one risk factor (STI, rough sex or chemsex including nasal or intravenous drug administrations) was identified in 72% of HIV-positive individuals and 83% of HIV-negative individuals. The use of intravenous drugs was reported by 5 out of 6 HIV-negative patients (83%), but in only 40% of HIV-positive patients. Thirteen reinfections were observed, all in HIV-positive patients (eradication occurred following spontaneous cure in 2 patients and post-treatment in 11).

**Table 1:** Characteristics of HIV-positive and HIV-negative MSM with HCV infection.

	HIV-infected (n = 43)	HIV-negative (n = 6)
**Age (median [IQR])**	48 [37.5–52.5]	39.5 [32.4–53.5]
**CDC**		NA
**A**	31	
**B**	6	
**C**	6	
**Antiretroviral treatment (n (%))**	42 (98%)	NA
**CD4 cells count (median [IQR])**	663 [564–790,5]	NA
**HIV-RNA <50 copies/mL (n (%))**	40 (93%)	NA
**Current STI (n (%))**	18 (42%)	2 (33%)
**IDU/ChemSex (n (%))**	18 (42%)	5 (83%)
**Rough sex (n (%))**	6 (14%)	2 (33%)
**STI, chemsex or rough sex (n (%))**	32 (74%)	5 (83%)
**HCV Genotype**		
**1a**	26	3
**4d**	17	3
**Reinfection (n (%))**	13 (30%)	0
**Engaged in PrEP (n (%))**	NA	1 (17%)

CDC: Centers for Disease Control and Prevention HIV category; STI: sexually transmitted infection; IDU: intravenous drug use; NA: non applicable.

During the study period, 47 out of the 49 patients infected with HCV were treated with direct-acting antivirals, from which 42 achieved sustained virologic response at week 12, 4 achieved end of treatment response and 1 was reinfected before week 12. One was lost to follow-up and 1 patient is awaiting treatment.

### Phylogenetic analyses

Based on NS5B sequencing, HCV strains identified in MSM patients during the study period belonged to either genotype 1a (n = 29) or genotype 4d (n = 20). In 78% of cases, sequencing was performed on a sample collected within 6 months after the estimated date of infection, *i*.*e*. during or near the acute phase of hepatitis C. In 4% of cases, the sample analysed was collected between 6 months and one year after the estimated date of infection and in 18% of cases, more than one year after the estimated date of infection.

**Fig 1:**
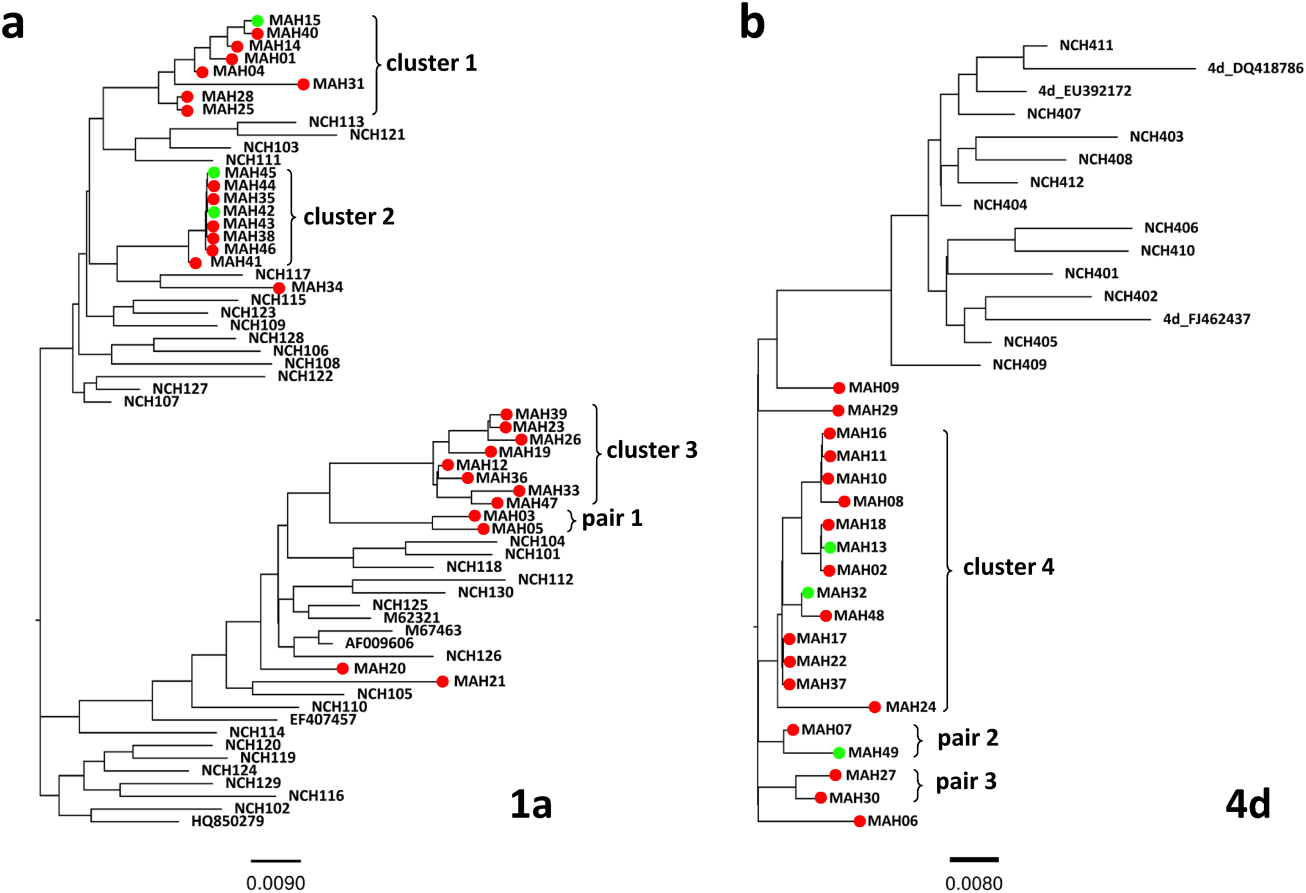
Phylogenetic trees of HCV NS5B sequences. Phylogenetic trees of NS5B sequences from HCV strains of genotype 1a (A) and 4d (B) isolated in this study including MSM and non-MSM patients. HCV sequences from HIV-positive and HIV-negative MSM are indicated by red and green circles, respectively. HCV sequences isolated in non-MSM patients and used for comparison are named NCH101-NCH120 for genotype 1a strains and NCH401-412 for genotype 4d strains. Reference strains for each subtype are identified by Genbank accession number. Identified transmission clusters and pairs are indicated by curly braces. Scale bars indicate nucleotide substitutions per site.

Phylogenetic trees were constructed with sequences from MSM and non-MSM patients (Fig 1). For genotype 1a, the 29 sequences from MSM clustered together with sequences from non-MSM patients in different branches of the phylogenetic tree. For genotype 4d, all 20 sequences from MSM clustered separately from sequences of non-MSM origin.

For genotype 1a, we identified three transmission clusters in MSM with eight isolates each and one pair. Notably, in cluster 2, seven of eight NS5B sequences had 100% homology. For genotype 4d, we identified one transmission cluster in MSM with 13 isolates and two pairs. Among all four clusters of more than 2 cases identified, clusters 1, 2 and 4 included isolates from both HIV-positive and HIV-negative, patients. Among the three pairs, pair 2 (genotype 4d) also included one HIV-positive and one HIV-negative patient.

### Chronological analysis of contagiousness

**Fig 2:**
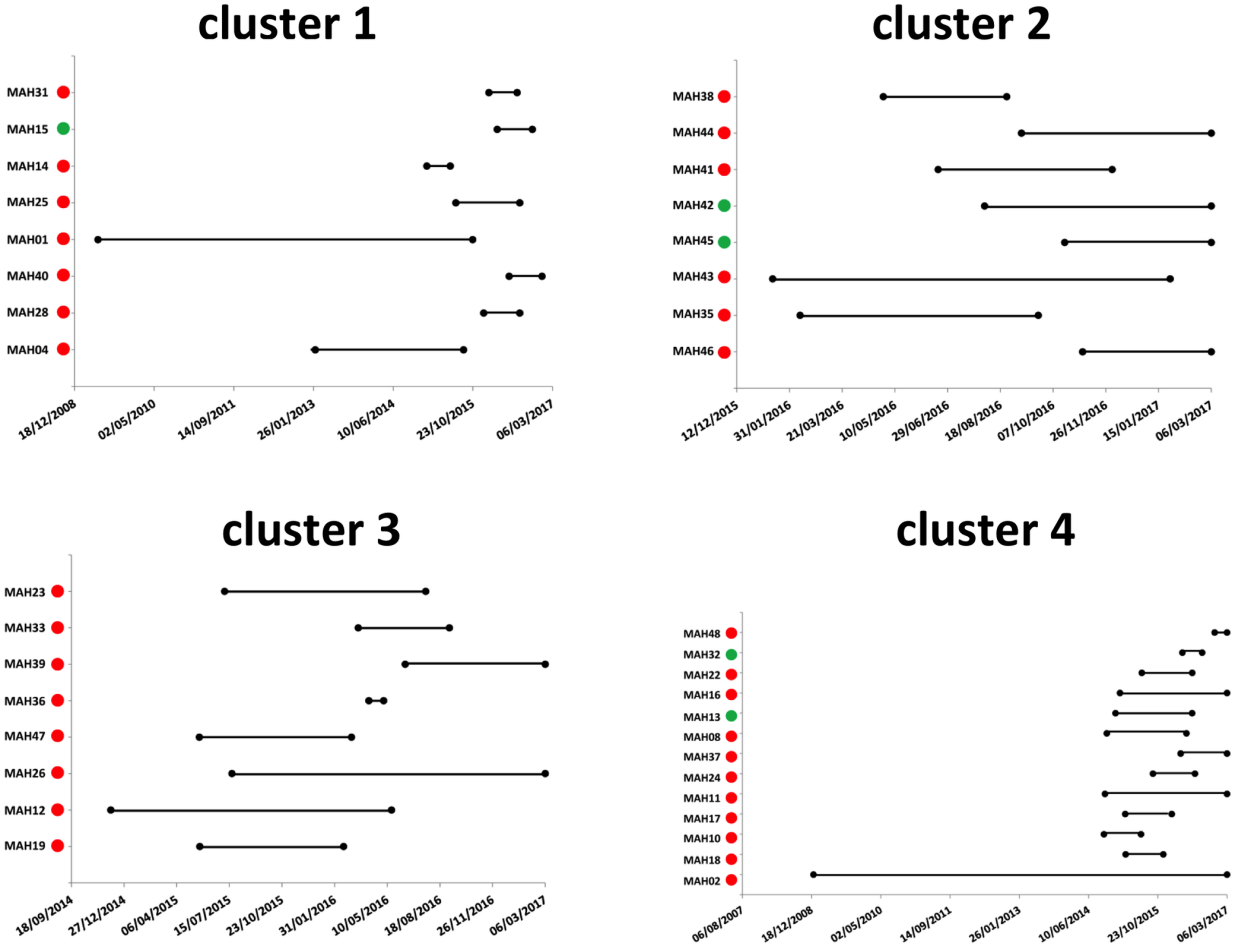
Individual periods of contagiousness within clusters of HCV-infected MSM. The period of contagiousness, as defined in the material and methods section, is represented by a horizontal black line. HIV-positive and HIV-negative MSM infected by HCV are indicated by red and green circles, respectively.

Based on the estimated time of HCV infection and the time of the last detectable HCV viral load during follow-up, we evaluated the periods of contagiousness for each member of the transmission clusters (Fig 2). In each cluster, individual periods of contagiousness overlapped, supporting the cluster identification. Each cluster mixing HIV-positive and HIV-negative MSM, started with several HIV-positive patients infected with HCV before the first HIV-negative patient was identified.

Characteristics of the patients included in the 4 clusters identified are presented in Table 2. Cluster 2 was characterized by a shorter interval between infections, the 8 acute HCV infections being diagnosed in less than 10 months (Fig 2). Additionally, patients in cluster 2 appeared younger than patients from the other clusters, and 5 out of 8 where using drugs, a higher proportion than in the other clusters. This period of contagiousness, together with the very high homology between the NS5B sequences within this cluster (Fig 1A), and with demographic and behavioural characteristics, suggests that direct transmission of HCV between members of this cluster may have occurred, presumably through drug use. In cluster 4, one HIV-positive patient was infected and remained viremic several years before other grouped cases were identified in a very short period, suggesting that this patient could be the source of the following cases.

**Table 2:** Characteristics of MSM within clusters of HCV infection.

	Cluster 1 (G1a) n = 8	Cluster 2 (G1a) n = 8	Cluster 3 (G1a) n = 8	Cluster 4 (G4d) n = 13
**Age (median [range])**	47 [37–62]	34 [26–63]	53 [48–64]	49 [28–69]
**HIV negative (n)**	1	2	0	2
**Engaged in PrEP (n)**	0	1	0	0
**Current STI (n)**	2	4	2	7
**IDU/ChemSex (n)**	2	5	3	6
**Rough sex (n)**	1	1	3	2
**STI, chemsex or rough sex (n)**	4	6	7	10
**Reinfection (n)**	1	1	5	4
**Interval between 1**^**st**^ **and last acute HCV (months)**	40.6	9.8	18.6	96.6

STI: sexually transmitted infection; IDU: intravenous drug use.

## Discussion

From a cohort of 49 HIV-positive and HIV-negative MSM patients with acute hepatitis C infection, we performed phylogenetic analyses of HCV strains based on NS5B sequencing and identified several clusters mixing HIV-positive and HIV-negative patients. Such clusters of HCV infections in MSM had been described for HIV-positive MSM only [3,9,26] but mixed clusters combining HIV-positive and HIV-negative MSM had been previously reported in only one case [27]. In our study, all HCV strains infecting HIV-negative MSM were included in clusters with strains also infecting HIV-positive MSM. Indeed, HCV sequences of HIV-negative MSM were highly similar to those of HIV-positive MSM, including one cluster showing nearly 100% homology between all sequences. Moreover, we identified two mixed clusters of genotype 1a as well as one mixed cluster and one mixed pair of genotype 4d. This suggests that transmission events of HCV between HIV-positive and HIV-negative MSM are not uncommon. This observation is supported by the chronological analysis of the individual periods of contagiousness. In all mixed clusters, HCV infections were first identified in HIV-positive MSM, indicating that these viruses were circulating in the HIV-positive population before their introduction in the HIV-negative one. This chronology of events was confirmed in cluster 4, where a single HIV-positive patient infected since several years, was suspected to be the common source of infection in this mixed cluster. However, as some personal data (such as the direct interactions between MSM in social venues) were not collected, the precise chains of HCV transmission could not be inferred in the clusters.

High-risk practices appeared quite similar in HIV-positive and HIV-negative MSM, suggesting that sharing these practices was the probable cause of infection in both groups. Additionally, a significant number of HCV infections in HIV-positive patients were actually reinfections, suggesting repeated high risk behaviours [28]. Since most HIV-positive patients had an undetectable HIV viral load, and exhibited therefore a limited contagiousness regarding HIV, while 4 out of 6 HIV-negative patients were PrEP users, one might suspect that serosorting giving up by HIV-negative MSM resulted in more HCV-related risk with HIV-positive patients. Interestingly, only 1 patient was receiving PrEP at the time of HCV diagnosis, while 3 infections were diagnosed in patients later enrolled in a PrEP program, indicating that high-risk practices precluded the use of PrEP in this population.

The spread of HCV in HIV-negative MSM may have significant implications in the future. Epidemiological data and modelling studies demonstrated that HCV infections will dramatically decrease in the HIV-infected population in France within the next years, due to a high treatment uptake and a low rate of new infections (Virlogeux et al., oral communication at CROI 2017 and [29]). Unlike HIV-positive MSM, for whom HCV screening is regularly performed, HIV-negative MSM are less followed for obvious reasons. Therefore, incidence of acute HCV in HIV-negative MSM is difficult to evaluate because the total number of individuals at risk of infection is unknown in this population. On-going monitoring for HCV infection among PrEP users and HIV-negative MSM in general is not clearly recommended in the guidelines [30,31]. Thus, underdiagnosed HCV infection in HIV-negative MSM can facilitate a cryptic reservoir, which might contribute to the expansion of HCV epidemic and may fuel again the HCV epidemic in high-risk HIV-positive MSM.

This study highlights that engagement in existing HCV screening guidelines among HIV-positive MSM remains crucial. In our study, acute HCV infections in HIV-negative MSM were mostly diagnosed either in patients receiving PrEP, or awaiting for PrEP enrollment, demonstrating the benefit of these programs for HCV testing in HIV-negative MSM. Implementation of routine HCV testing among all HIV-negative MSM with high-risk practices should be included in the guidelines. To improve future control of the HCV epidemic in MSM, it appears essential to identify patients with high-risk practices regarding HCV and to engage them in prevention interventions including risk reduction and HCV screening.

## Supporting information

S1 Table Genbank accession numbers of sequences reported in the study. (DOCX)
