# Supplementary material for: Hepatitis C virus spread from HIV-positive to HIV-negative men who have sex with men
Source: PLoS One. 2018 Jan 2;13(1):e0190340. doi: 10.1371/journal.pone.0190340 (PMC5749770; doi:10.1371/journal.pone.0190340)
Supplement: S1 Table — (DOCX) [file pone.0190340.s001.docx]

Table S1. Genbank accession numbers of sequences reported in the study.

|  | Sequence name | Genbank accession number |
| --- | --- | --- |
| MSM patients | MAH01 | KY928311 |
|  | MAH02 | KY928312 |
|  | MAH03 | KY928313 |
|  | MAH04 | KY928314 |
|  | MAH05 | KY928315 |
|  | MAH06 | KY928316 |
|  | MAH07 | KY928317 |
|  | MAH08 | KY928318 |
|  | MAH09 | KY928319 |
|  | MAH10 | KY928320 |
|  | MAH11 | KY928321 |
|  | MAH12 | KY928322 |
|  | MAH13 | KY928323 |
|  | MAH14 | KY928324 |
|  | MAH15 | KY928325 |
|  | MAH16 | KY928326 |
|  | MAH17 | KY928327 |
|  | MAH18 | KY928328 |
|  | MAH19 | KY928329 |
|  | MAH20 | KY928330 |
|  | MAH21 | KY928331 |
|  | MAH22 | KY928332 |
|  | MAH23 | KY928333 |
|  | MAH24 | KY928334 |
|  | MAH25 | KY928335 |
|  | MAH26 | KY928336 |
|  | MAH27 | KY928337 |
|  | MAH28 | KY928338 |
|  | MAH29 | KY928339 |
|  | MAH30 | KY928340 |
|  | MAH31 | KY928341 |
|  | MAH32 | KY928342 |
|  | MAH33 | KY928343 |
|  | MAH34 | KY928344 |
|  | MAH35 | KY928345 |
|  | MAH36 | KY928346 |
|  | MAH37 | KY928347 |
|  | MAH38 | KY928348 |
|  | MAH39 | KY928349 |
|  | MAH40 | KY928350 |
|  | MAH41 | KY928351 |
|  | MAH42 | KY928352 |
|  | MAH43 | KY928353 |
|  | MAH44 | KY928354 |
|  | MAH45 | KY928355 |
|  | MAH46 | KY928356 |
|  | MAH47 | KY928357 |
|  | MAH48 | KY928358 |
|  | MAH49 | KY928359 |
| Non-MSM patients | NCH101 | KY928360 |
|  | NCH102 | KY928361 |
|  | NCH103 | KY928362 |
|  | NCH104 | KY928363 |
|  | NCH105 | KY928364 |
|  | NCH106 | KY928365 |
|  | NCH107 | KY928366 |
|  | NCH108 | KY928367 |
|  | NCH109 | KY928368 |
|  | NCH110 | KY928369 |
|  | NCH111 | KY928370 |
|  | NCH112 | KY928371 |
|  | NCH113 | KY928372 |
|  | NCH114 | KY928373 |
|  | NCH115 | KY928374 |
|  | NCH116 | KY928375 |
|  | NCH117 | KY928376 |
|  | NCH118 | KY928377 |
|  | NCH119 | KY928378 |
|  | NCH120 | KY928379 |
|  | NCH121 | KY928380 |
|  | NCH122 | KY928381 |
|  | NCH123 | KY928382 |
|  | NCH124 | KY928383 |
|  | NCH125 | KY928384 |
|  | NCH126 | KY928385 |
|  | NCH127 | KY928386 |
|  | NCH128 | KY928387 |
|  | NCH129 | KY928388 |
|  | NCH130 | KY928389 |
|  | NCH401 | KY928390 |
|  | NCH402 | KY928391 |
|  | NCH403 | KY928392 |
|  | NCH404 | KY928393 |
|  | NCH405 | KY928394 |
|  | NCH406 | KY928395 |
|  | NCH407 | KY928396 |
|  | NCH408 | KY928397 |
|  | NCH409 | KY928398 |
|  | NCH410 | KY928399 |
|  | NCH411 | KY928400 |
|  | NCH412 | KY928401 |
